# Metastasis to the occipitocervical junction: A case report and review of the literature

**DOI:** 10.4103/2152-7806.63911

**Published:** 2010-05-31

**Authors:** Risheng Xu, Daniel M. Sciubba, Ziya L. Gokaslan, Ali Bydon

**Affiliations:** 1 Medical Scientist Training Program, Johns Hopkins School of Medicine, Baltimore, Maryland; 2 Department of Neurosurgery, Johns Hopkins School of Medicine, Baltimore, Maryland; 3 Johns Hopkins Spinal Column Biomechanics and Surgical Outcomes Laboratory, Baltimore, Maryland

**Keywords:** Cervical, craniocervical, instability, metastasis, occipital

## Abstract

**Background:**

The management of metastatic spinal disease is generally considered palliative, as the progression of systemic disease is likely to hinder survival. Although the occurrence of C1-C2 instability due to metastatic disease is not uncommon and thus treatment options have been well-defined, craniocervical instability due to lesions occurring at the junction of the occiput and atlas is more rare, and treatment for metastasis to this region is less well-defined.

**Case Description:**

We present a patient with non-small-cell lung cancer metastatic to the atlanto-occipital facet joint complex. A drastic improvement in the presenting debilitating mechanical neck pain was noted following an occipitocervical fusion. A literature review of published cases of metastases to the occipitocervical junction was conducted along with treatment options.

**Conclusions:**

The atlanto-occipital facet joint is a rare site of metastatic disease. Destruction of this joint can lead to significant neck pain secondary to instability. Spinal fusion may afford significant and rapid resolution of these symptoms, and should be considered in the management of patients—even those with end-stage oncologic disease.

## INTRODUCTION

Lung cancer is classified by the World Health Organization as the leading cause of cancer-related deaths in men and the second highest in women, claiming more than 1.2 million lives worldwide per year.[26] In the United States alone, nearly 215,000 new cases of lung cancer will be diagnosed this year; 161,000 will succumb to the disease.[20,21] This translates to lung cancer accounting for almost 30% of all cancer mortality in the United States, more than breast, prostate, and colon cancer combined. Of all lung cancers—diagnosed via histopathological criteria—non-small-cell (NSCLC) type accounts for approximately 80% of all primary lung tumors.[24]

The prognosis of patients diagnosed with lung cancer is poor, due to association with systemic, incurable disease.[10] As of 2005, although the average age of presentation of newly diagnosed patients increased to approximately 68 years, 65% were initially diagnosed with Stage IIIB or IV disease, with a median survival rate of four to six months.[22] Less than 20% of patients remained alive at one year.

NSCLC metastasizes to a variety of locations within the body, including the brain, bone, liver, and adrenal glands, in order of decreasing prevalence.[19] Of note, the skeleton is a particularly common site of metastasis, with most recent retrospective studies estimating the incidence of NSCLC skeletal metastases to be 24% in American populations,[13] and as high as 30% among the Japanese.[25] Although NSCLC appears to metastasize most frequently to the axial skeleton, the involvement of the craniovertebral junction is far rarer.

In this report, we present a patient with known NSCLC metastasis who presented with severe axial neck pain due to tumor infiltration and destruction of the atlanto-occiptal joint. Although the etiologies and treatment strategies for managing destructive lesions of the atlanto-axial (C1-C2) joint are known, to our knowledge, this is the first published case of an NSCLC lesion that metastasized to both the occiptal condyle and the atlas (atlanto-occipital joint), causing instability and severe neck pain. We also review the literature and discuss current treatment options for bony metastasis to the occipitocervical junction.

## CLINICAL SUMMARY

**Figure 1:**
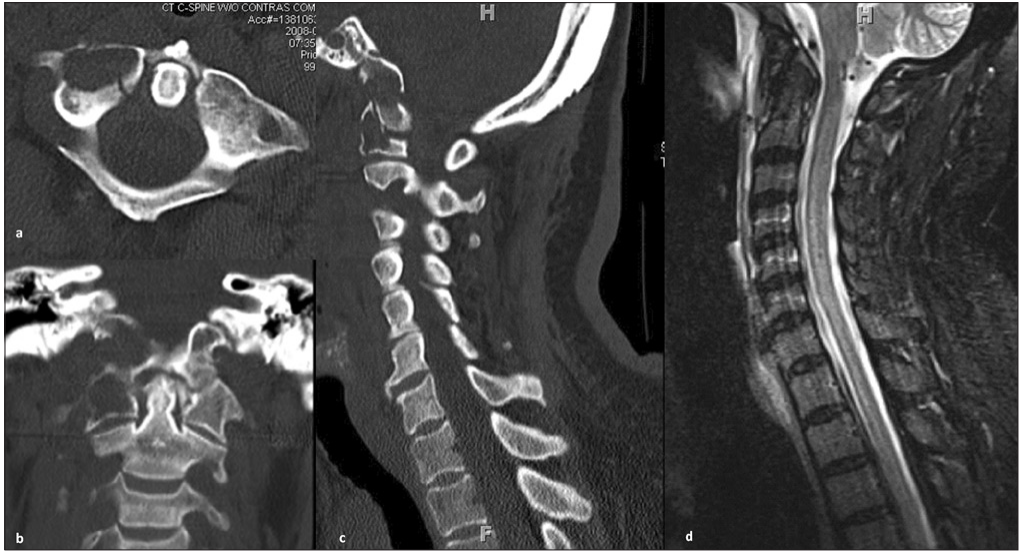
Computed tomography (CT) and magnetic resonance imaging (MRI) of the atlanto-occipital junction. a. An axial CT image shows significant hypodense areas in the right occipitocervical junction, demonstrating extensive tumor infiltration. b. The coronal CT image again illustrates the scope of metastatic disease in both the right atlas and occipital condyle, with both being almost entirely consumed by the tumor. c. A sagittal view shows hypodense destructive lytic masses in both the occipital condyle and atlas. d. A T2-weighted MR image shows normal cerebral spinal fluid distribution with no evidence of spinal cord compression

A 66-year-old female with a two-year history of NSCLC status post lobectomy, with metastasis to the right kidney, right femur and pelvis, presented with severe occipital headaches and debilitating neck pain. She was functionally independent, with a Karnofsky score of 90. She was neurologically intact except for a left homonymous hemionopsia due to an old posterior cerebral artery distribution infarct. Brain and cervical spine computed tomography (CT) and magnetic resonance imaging (MRI) scans revealed an old right occipital stroke, right C1 lateral mass and occipital condyle destructive lytic lesions, and a fracture of the right atlanto-occipital joint [Figure 1a–c]. There was no evidence of spinal cord compression [Figure 1d]. She had difficulty sitting up or standing up due to severe axial neck pain. She was started on intravenous morphine and placed in a cervical collar, which improved her pain minimally. Due to the destruction of the joint evident on radiographic images and the partial relief of the pain after stabilizing her in a neck orthosis, it was thought that the patient would benefit from internal stabilization.

**Figure 2:**
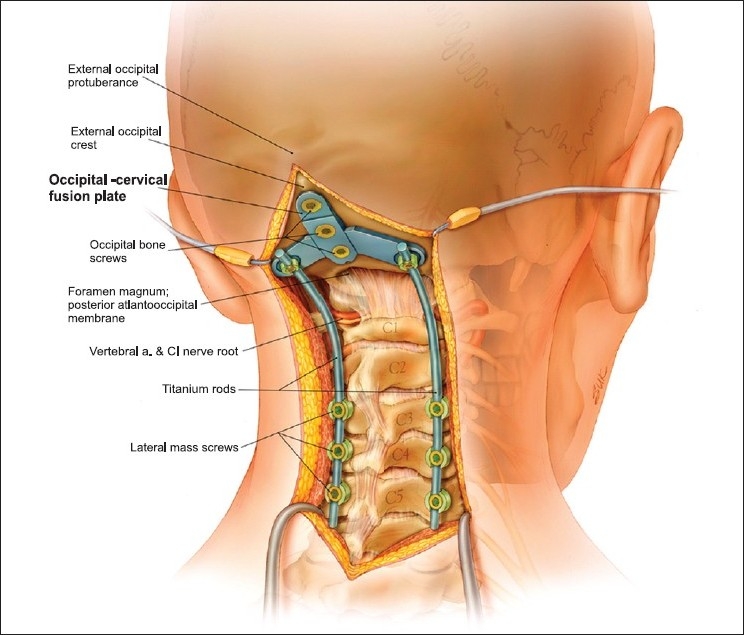
A 3-D illustration shows the postoperative reconstruction and stabilization of the occipitocervical region using an occipital plate and lateral mass screws at C3, C4, and C5. © Ian Suk, Department of Neurosurgery, Johns Hopkins School of Medicine

**Figure 3:**
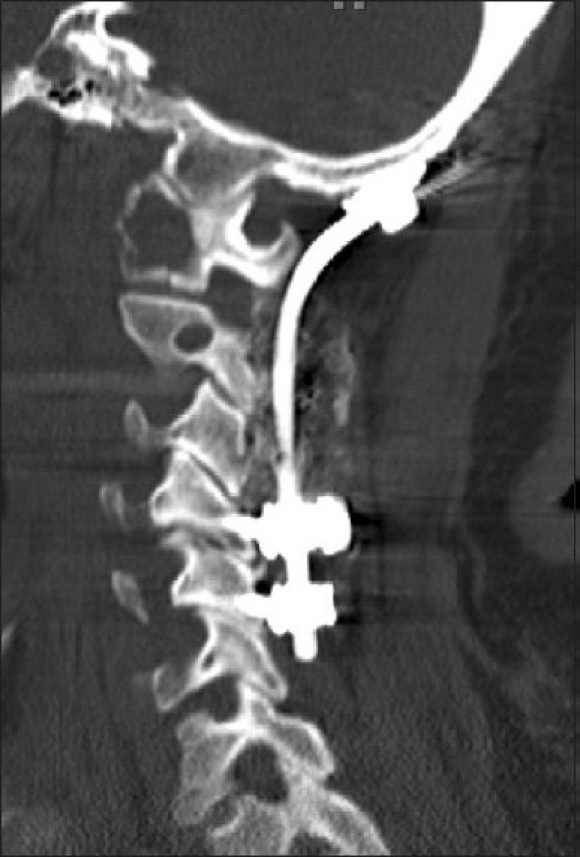
A postoperative sagittal cervical spine CT shows the placement of occiput to C5 instrumentation. (Note that the metastatic NSCLC lesion at the C0-C1 joint was not resected during surgery and that the subsequent alleviation of pain in the patient was achieved through mechanical stabilization of the occipitocervical joint alone)

**Figure 4:**
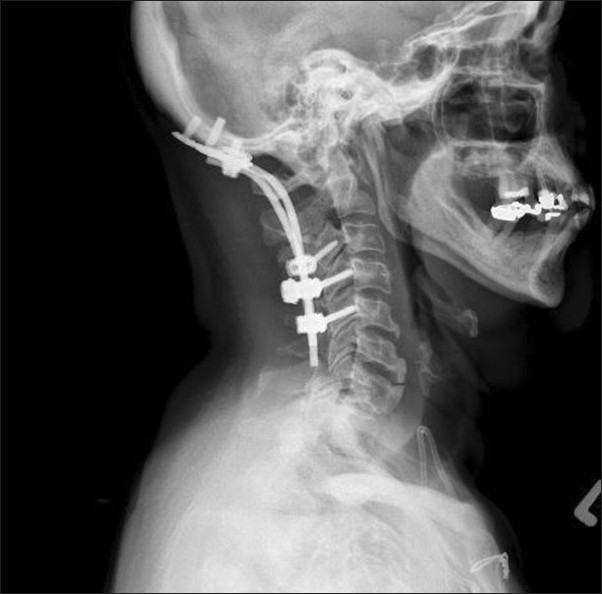
A five-month postoperative lateral X-ray image of the cervical spine shows intact instrumentation without any evidence of hardware loosening, pullout, or failure

The patient underwent an occiput to C5 instrumented stabilization and fusion using an occipital plate connected to lateral mass screws at C3, C4, and C5 [Figures 2 and 3]. Demineralized bone matrix (Optium, DePuy, Johnson and Johnson), mixed with cancellous bone chips were placed over the arthrodesed lamina and joints. Postoperatively, she experienced substantial neck pain relief, and was discharged in stable neurologic condition. She remained independently ambulatory and resided with her family. At her most recent follow-up (12 months postoperatively), the patient exhibited a neck pain level of 2/10, demonstrating sustained pain relief. A lateral cervical spine X-ray revealed no evidence of hardware failure or kyphosis [Figure 4]. The patient succumbed to her metastatic disease 14 months postoperatively.

## DISCUSSION

Distant bony metastases of lung cancer have been associated with significant skeletal-related events (SREs), including pain (secondary to either tumor infiltration or ensuing mechanical instability), pathological fractures, hypercalcemia, and spinal cord compression.[4] Of these comorbidities, bone pain is the most common, seen in 80% of patients with skeletal NSCLC metastases.[6,12] Other SREs such as fractures and hypercalcemia owe their pathophysiology primarily to the tumor-induced increase of osteoclast differentiation relative to osteoblasts.[8,11] Nevertheless, despite the prevalence of skeletal metastases—with the spine, ribs, and pelvis being the most common—encroachment upon the occipital condyle and atlas is exceedingly rare. Within the English literature (Pubmed/Medline), no case of NSCLC metastasis to the occipitocervical junction has been previously described.

Metastatic involvement of the craniovertebral junction due to other primary tumors has been described in seven cases with documentation of involvement of the C0-C1 junction and ensuing instability [Table 1]. Specifically, George *et al*. reported four cases of metastatic cancer involving the atlanto-occiptal joint over a 20-year period from 1981-2001.[9] In a case report, Akai *et al*. described a patient with malignant fibrous histiocytoma invasion of the right craniovertebral joint who presented with intractable occipitalgia.[2] Elia *et al*. described a patient with C0-C1 instability due to myeloma,[7] and Laohacharoensombat *et al*. described a case of metastatic follicular thyroid cancer to the occipitocervical region.[14] Thus, to our knowledge, this is the first reported case in the English literature, of NSCLC metastasis to the craniovertebral junction causing instability of the occipito-atlantal joint and subsequent severe axial neck pain.

**Table 1:** Summary of other metastatic occipitocervical tumors, their treatments, and their post-operative outcomes

Reference	Year	Sex	Age	Indication for Operation	Pathology	Treatment	Outcome at Last Followup
Elia et al	1990	F	70	C0-C1 instability	Myeloma	C0-C4 Fusion using the Onlay technique	Fused at 16 weeks
Laohacharoensombat *et al*	1990	F	43	Neck pain and radiculopathy; tumor invasion of foramen magnum with atlanto-occipital subluxation; C3, C5 tumor invasion	Follicular thyroid carcinoma	C0-C7 fusion using an occipital pin with additional cement reinforcement	Neck stiffness, ambulation restored, lost to follow up at 2-3 months
Akai *et al*	2006	F	59	Severe occipitalgia, right C0-C1 joint destruction	Malignant fibrous histiocytoma	Partial tumor resection, occipitocervical fusion with the Olerud Cervical Fixation System, post-operative radiation therapy and chemotherapy	Resolution of headaches; tumor recurrence followed by resection two additional times; patient became paraplegic and died of respiratory failure; 2 years and 4 months total treatment course
George *et al*	2006	F	46	Upper neck pain, C0-C1 tumor invasion, lateral condyle destruction	Melanoma	Lateral approach, occipitocervical fusion with plates and bone graft	Fusion achieved, significant pain resolution
George *et al*	2006	M	57	Upper neck pain, C0-C1 tumor invasion, lateral condyle destruction	Thyroid	Tumor embolization, lateral approach, occipitocervical fusion with plates and bone graft	Fusion achieved, significant pain resolution
George *et al*	2006	M	63	Upper neck pain, C0-C1 tumor invasion, lateral condyle destruction, tetraplegia	Lung (pathology unknown)	Lateral approach, occipitocervical fusion with plates and bone graft	Respiratory failure due to extensive metastasis leading to death
George *et al*	2006	M	61	Upper neck pain, C0-C1 tumor invasion, lateral condyle destruction	Thyroid	Tumor embolization, lateral approach, occipitocervical fusion with plates and bone graft	Fusion achieved, significant pain resolution

Given the poor prognosis of metastatic spine disease, especially late-stage NSCLC, treatment for such lesions are palliative in nature. Well-defined NSCLC lesions may be treated with radiotherapy and/or surgery.[1] Specifically, surgical intervention is generally indicated for patients with symptomatic epidural spinal cord compression, spinal column instability leading to pain or neurological deficits, or radio-resistant tumors in patients with life expectancies of at least six months duration.[16] Concurrent systemic disease is simultaneously treated with chemotherapeutic agents, hormonal therapy, bisphosphonates, and radionucleotides.[3,17] Although double-regimen, platinum-based chemotherapeutics are the standard of care for treating advanced NSCLC, recent Phase III clinical trials are still establishing the efficacies of new multimodal agents based on novel cytotoxic and target-based therapeutics. [5,20,23] In addition, current Phase II clinical trials utilizing monoclonal antibodies to inhibit osteoclast function have revealed promising data as an alternative method to suppressing osteoclast activity in mediating bone loss/resorption and hypercalcemia compared with bisphosphonates, currently considered the standard of care. [15]

Although survival of patients with metastatic spinal disease ultimately depends on the extent and control of the underlying systemic disease, quality of life can be substantially improved with surgical stabilization. In their landmark study, Patchel *et al*. concluded that direct surgical decompression plus radiation therapy for metastatic epidural spinal cord compression improved functional status, relative to ambulation and continence, compared to radiation alone.[18] Although some patients in that series suffered neurological decline secondary to spinal cord compression, we believe it is likely that surgical stabilization also contributed substantially to the improvement in postoperative ambulation due to the treatment of mechanical instability and its associated pain. In our patient, such mechanical pain was drastically improved with minimal surgical morbidity. Of note, although sectioning of the C2 nerve roots may be an effective method for controlling pain in patients with C2 neuralgia, it was felt that in this patient′s case her debilitating neck pain was due mainly to the destruction of her atlanto-occipital joint; thus, open C2 rhizotomy was not performed. In reviewing the seven other published cases of metastatic disease to the occipitocervical junction, all treatments have also consisted of occipitocervical fusion, coupled with tumor resection, radiation, and/or chemotherapy.[2,7,9,14] In our case, fixation points at C1 and C2 were not introduced as we felt that strong bicortical fixation points were achieved in the occiput, as well as at C3, C4, and C5.

In most cases, patients with metastatic disease at the atlanto-occipital joint experience a reduction in headache or upper neck pain following occipitocervical fusion.[2,7,14] However, it was noted that preoperative motor loss and cranial nerve deficits such as swallowing difficulties or hoarseness were not restored postoperatively.[18] As a result, in those patients with significant preoperative neurological deficits in addition to mechanical pain, expectations following surgical treatment should be carefully addressed with patients with regards to likelihood of improved neurological function. Currently in the United States, the general practice is to offer occipitocervical fixation to patients with a life expectancy greater than three to six months. As healthcare policies in the United States continue to evolve, it is hoped that the decision on whether to pursue an operative fusion procedure in the face of metastatic disease will remain a choice made jointly by the patient, his/her treating oncologist and neurosurgeon.

## CONCLUSIONS

This is the first reported case of NSCLC metastasizing to the atlanto-occipital joint causing severe sudden onset neck pain due to mechanical instability. Significant alleviation of pain was achieved via surgical stabilization (occipitocervical fusion). Although metastatic disease caused by NSCLC carries a poor prognosis, occipitocervical stabilization may provide excellent pain relief and improvements in quality of life in properly selected patients.
